# S181. THE STATE OR TRAIT COMPONENT OF DOPAMINE AND GLUTAMATE DYSFUNCTION IN THE RISK FOR PSYCHOSIS: AN IN VIVO MULTIMODAL IMAGING STUDY OF INDIVIDUALS WITH 22Q11.2 DELETION

**DOI:** 10.1093/schbul/sby018.968

**Published:** 2018-04-01

**Authors:** Maria Rogdaki, Mattia Veronese, Pamela Hathway, Sameer Jauhar, Maria Gudbrandsen, Eileen Daly, Oliver Howes

**Affiliations:** 1 MRC London Institute of Medical Sciences, Imperial College; 2 Center for Neuroimaging Science, Institute of Psychiatry, Psychology and Neuroscience, King’s College London; 3 Imperial College; 4 Institute of Psychiatry, Psychology & Neuroscience, King’s College London; 5 MRC London Institute of Medical Sciences, King’s College London

## Abstract

**Background:**

Dopaminergic and glutamatergic dysregulation are among the leading hypotheses for schizophrenia. Previous in vivo imaging studies have shown that increased striatal presynaptic dopamine synthesis capacity (DSC) predates the onset of psychosis and is associated with symptom severity. Recent meta-analysis in magnetic resonance spectroscopy studies in schizophrenia reported increased levels of glutamate and glutamine (Glx) in basal ganglia and medial frontal cortex in clinical high-risk groups for psychosis. Despite the evidence of alterations in both dopamine and glutamate in schizophrenia, the degree to which these alterations are trait markers linked to genetic risk for psychosis or reflects state changes is not clear from previous studies. Over the last fifteen years, it has been well established that 22q11 deletion is one of the most important genetic risk factors for the development of schizophrenia. Individuals with 22q11 deletion are at increased genetic risk for psychosis, reaching a prevalence 30% for psychotic disorder. The aims of our study were to investigate dopaminergic and glutamatergic function in individuals with 22q11.2 deletion.

**Methods:**

Participants underwent 18F DOPA PET, MRI, as well as clinical measures.21 individuals with 22q11 deletion (14 females and 7 males, age (mean, SD): 26.1(7.72)) and 26 healthy volunteers (15 females and 11 males, age (mean, SD): 26.12(4.28)) took part in the 18F DOPA PET. Standardised ROIs was defined in the striatum, including limbic, associative and sensorimotor sub-regions, and the reference region, defined according to previous study. The ROI atlas was normalised to each individual PET dynamic image. A Patlak analysis was applied to calculate influx constants (Ki values) for the whole striatal ROI relative to uptake in the cerebellar reference region (Kicer [min-1]).

In addition, 17 individuals with 22q11 deletion (11 females and 6 males, age (mean, SD): 26.39(7.7)) and 30 healthy controls (17 females and 13 males, age (mean, SD): 27.17(4.8)) had MRI. 1H-MRS voxels were placed on the anterior cingulate cortex and left striatum. Spectra were analyzed using LC Model version 6.3-1L. Poorly fitted metabolite peaks (Cramer–Rao minimum variance bounds >20% as reported by LC Model) were excluded from further analysis.

**Results:**

DSC in the whole striatum was significantly increased in the individuals with 22q11 deletion compared to healthy controls (mean (Kicer [min-1] =0.0143; SD=0.001; mean Kicer [min-1] = 0.0127; SD=0.001, respectively; effect size (Cohen’s d= 1.47, p< 0.000). In addition, no difference was found between groups in Glx levels in anterior cingulate cortex (individuals with 22q11 deletion; mean=19.85; SD=3.17, healthy controls; mean= 21.04; SD=3.91) and left striatum (individuals with 22q11 deletion; mean=11.45; SD=2.88, healthy controls; mean=11.6;SD=2.74)(t(44) =-1.065; p=0.29, t(42)=-0.172;p=0.86, respectively).Psychopathology scales were not correlated with either dopaminergic or glutamatergic function in the group of 22q11 deletion.

**Discussion:**

Our findings provide evidence that dopamine synthesis capacity has a strong trait component and glutamatergic dysfunction may be most likely associated with disease status. Future studies with longitudinal design are warranted to further investigate the role of dopamine and glutamate in individuals with 22q11 deletion. Moreover, there is a clear need for studies utilizing higher field resonance strength and appropriate radiotracers to investigate glutamatergic function in this population. These will be crucial steps towards the development of new treatments, that can be applied in early stages of illness.

